# Low complication and high bone union rates following acute oblique fibular osteotomy with suture ligation prior to closing wedge high tibial osteotomy: A multicenter study of 231 cases

**DOI:** 10.1002/jeo2.70779

**Published:** 2026-05-25

**Authors:** Dai Sato, Kazunori Yasuda, Eiji Kondo, Shuken Kai, Koji Yabuuchi, Jun Onodera, Norimasa Iwasaki, Tomonori Yagi

**Affiliations:** ^1^ Department of Orthopaedic Surgery Yagi Orthopaedic Hospital Sapporo Japan; ^2^ Department of Orthopaedic Surgery Hokkaido University Graduate School of Medicine Sapporo Japan; ^3^ Center for Sports Medicine, Hokkaido University Hospital Sapporo Japan; ^4^ Department of Orthopaedic Surgery Higashi Hokkaido Hospital Kushiro Japan

**Keywords:** bone union, complications, fibular osteotomy, high tibial osteotomy, lateral closing wedge

## Abstract

**Purpose:**

Fibular shortening is required in lateral closing wedge (LCW) high tibial osteotomy (HTO), yet the safety and healing profile of acute fibular oblique osteotomy with suture ligation (AOOL) remain unclear. This study evaluated complications, union rate, and time to union after AOOL, and identified factors associated with delayed union.

**Methods:**

A retrospective multicenter study was conducted on 240 medial osteoarthritic knees from 227 patients who underwent the AOOL procedure in combination with inverted V‐shaped (hemi‐LCW) HTO. Of the 227 patients, 231 knees from 218 patients were followed up for 2–4 years. Radiographs were taken at 2, 3, 6, 9 and 12 months postoperatively, as well as at the final follow‐up. Bone union was evaluated using a radiographic scoring system. Surgeries were performed by four surgeons. Prior to cohort pooling, it was verified that patient groups across the four surgeons showed no significant differences in patient characteristics, procedures, or outcomes. To present the complication and non‐union rates, two‐sided exact 95% confidence intervals for the proportions were calculated using the Clopper–Pearson method. Logistic regression analyses were performed to identify factors that significantly delay bone healing.

**Results:**

The complication rate was 1.7% (exact 95% confidence interval, 0.5%–4.4%). The non‐union rate was 1.3% (0.3%–3.7%). Accordingly, the bone union rate was 98.7% (96.3%–99.7%). The time to union was 7.0 months, with the standard deviation of 2.4. Significant factors associated with a longer time to achieve complete bone union at the fibular osteotomy site included male sex, lower bone mineral density, higher Kellgren–Lawrence grade, and a greater initial osteotomy gap.

**Conclusion:**

Among the 231 fibular osteotomies performed using the AOOL procedure, complications were rare, and the bone union rate was remarkably high. However, the average time to union was 7 months, which was considered relatively prolonged.

**Level of Evidence:**

Level II, cohort study.

## Graphical abstract

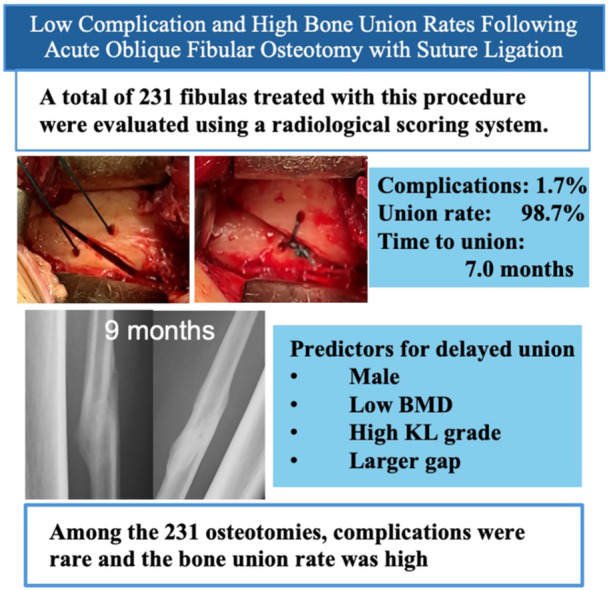

**Abbreviations**
ANOVA: one‐way analysis of variance
AOOL: acute oblique osteotomy and suture ligation
AP: anteroposterior
FTA: lateral femorotibial angle
HKA: hip‐knee‐ankle angle
HTO: high tibial osteotomy
iVHTO: inverted V‐shaped high tibial osteotomy
JOA: Japanese Orthopaedic Association
KOOS: Knee Injury and Osteoarthritis Outcome Score
LCW: lateral closing wedge
MOW: medial opening wedge
MPTA: medial‐proximal tibial angle
OA: medial osteoarthritis
PTFJ: proximal tibiofibular joint
RUSF: radiographic union score for fibular osteotomy
RUST: radiographic union score for tibial fractures
%MA: ratio of the point at which the mechanical axis passed across the joint line to the width of the tibial plateau

## INTRODUCTION

Lateral closing wedge (LCW) high tibial osteotomy (HTO) is a useful surgical option for treating medial compartment osteoarthritis of the knee with severe varus deformity. However, one disadvantage of LCWHTO is the need for an additional fibular osteotomy to shorten the fibula [11, 13]. An ideal fibular shortening procedure should be technically simple, free of complications or adverse events, consistently achieve bone union at the osteotomy site, and not interfere with the intended alignment correction in HTO [22]. However, to date, no fibular osteotomy technique has been reported that satisfies all of these criteria.

For example, the most common procedure to shorten the fibula is resecting an approximately 1 cm–long shaft at the central portion of the fibula [4, 7]. In this procedure, however, the incidence of peroneal nerve palsy has been reported to range from 2.8% to 13.7% [9, 15]. In addition, the incidence of non‐union at the fibular osteotomy site has been reported to range from 14% to 65% [3, 9, 15]. On the other hand, fibular head osteotomy procedures and proximal tibiofibular joint (PTFJ) release procedures have gained attention, as they avoid the risk of non‐union at the fibular shaft [5, 9, 14, 18, 20]. However, peroneal nerve palsy and pain at the osteotomy site continue to be reported following fibular head osteotomy procedures [3, 7], while progression of tibiofibular joint arthritis and increased varus instability of the knee have been identified as new complications following PTFJ release procedures [14, 18, 20]. Thus, there is a need to develop a new fibular osteotomy procedure that addresses all these requirements.

**Figure 1:**
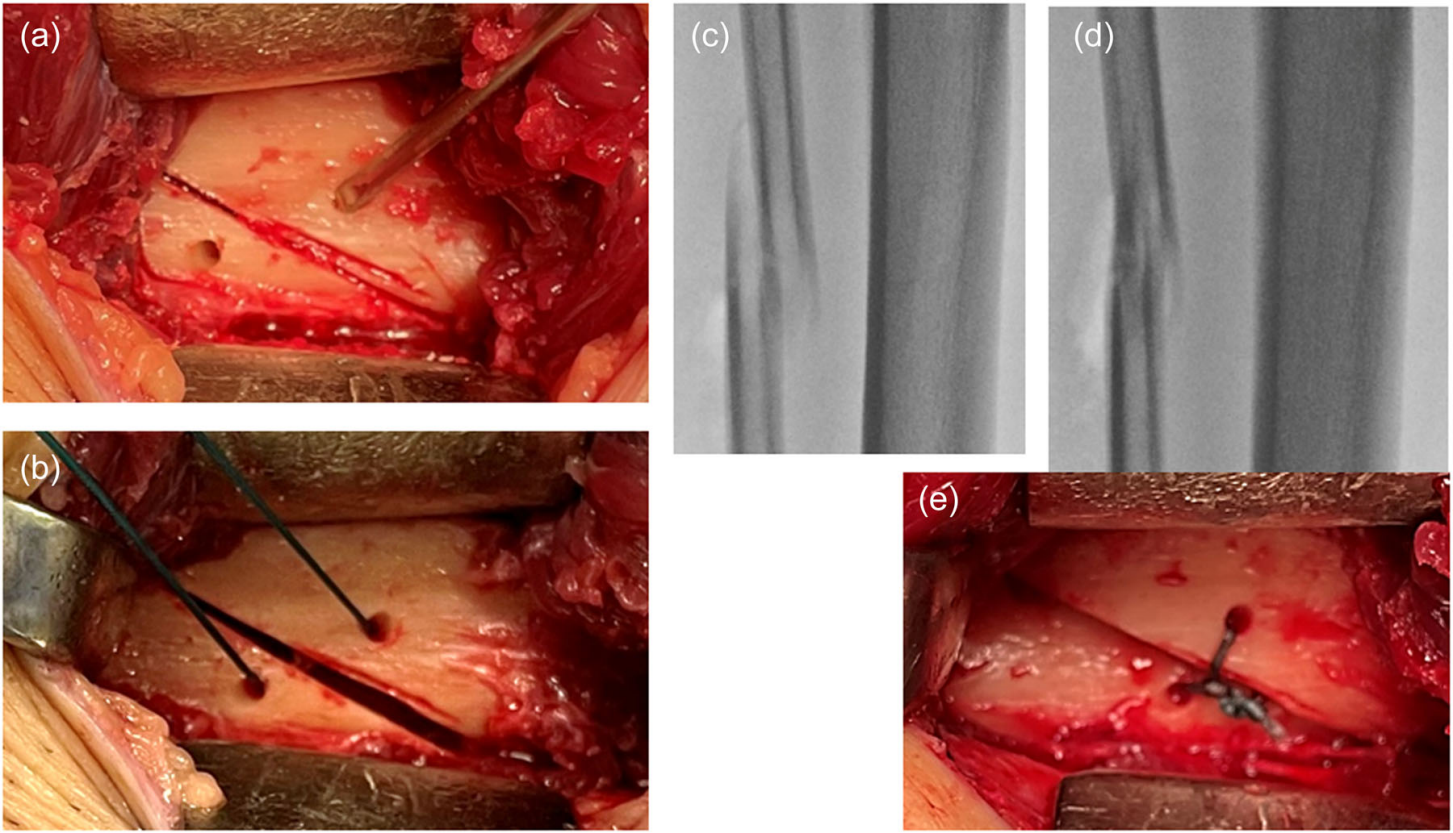
Acute oblique osteotomy and suture ligation (AOOL) procedure. (a) An acute oblique osteotomy was performed on the fibular shaft, and two thin holes were created at a point 3 mm from the osteotomy line. (b) A polyester thread was passed through the two holes. (c) When the high tibial osteotomy (HTO) was completed, the ends of the osteotomized fibula were displaced. (d) These fibular ends were reduced. (e) The polyester thread was securely tied.

In 2020, Yasuda et al. [26] reported a novel fibular shortening technique, termed the acute oblique osteotomy and suture ligation (AOOL) procedure. In this procedure, a simple oblique osteotomy in the frontal plane is performed at the central portion of the fibular shaft. Following tibial correction by HTO, the two ends of the osteotomy site are brought into contact and ligated with a polyester thread (Figure 1). In 2022, Ueda et al. [21] followed up 42 knees that underwent fibular osteotomy with AOOL procedure for 2 years postoperatively. They reported rarity of post‐operative complications and a fibular union rate of 97.6% following the AOOL procedure. In this study, however, the number of patients was too small to draw definitive conclusions regarding the fundamental characteristics of this procedure. Therefore, the safety and the healing capacity of the AOOL procedure have not yet been established. Moreover, the clinical course of bone healing after fibular osteotomy remains poorly understood. For example, the time to complete bone union at the osteotomy site and the factors affecting the bone union time have not been clarified.

Therefore, this study was conducted using a larger cohort, consisting of 240 knees from 227 patients. The first aim of this study was to evaluate the complication rate, the bone union rate at the osteotomy site, and the time to complete bone union following the AOOL procedure. The second aim was to investigate the factors that may delay the bone union time at the fibular osteotomy site, using univariate and multivariate logistic regression analyses.

## METHODS

### Study design

The study protocol was approved by the ethics review boards of all three participating institutions (Hospital I: No. H28‐0001, Hospital II: No. 018‐0213, Hospital III: No. 2018‐0001). All patients were informed about the use of their clinical data regarding surgical treatment for clinical research purposes, and consent was obtained from all patients.

**Figure 2:**
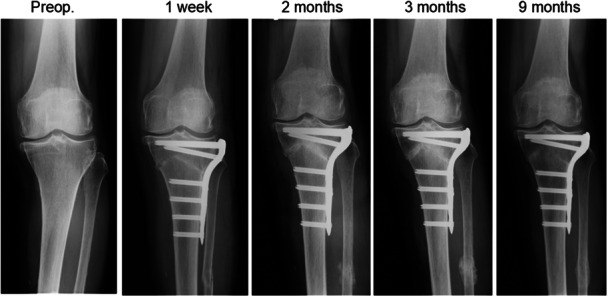
Inverted V‐shaped HTO with fibular osteotomy (AOOL procedure). In this case (62‐years‐old female), the medial proximal tibial angle was corrected by 14°. AOOL, acute oblique osteotomy and suture ligation; HTO, high tibial osteotomy.

A retrospective multicenter study was conducted on a total of 240 medial OA knees from 227 patients who underwent fibular osteotomies using the AOOL procedure in combination with inverted V‐shaped high tibial osteotomy (iVHTO). The iVHTO is defined as a combination of hemi‐LCW and hemi‐medial opening wedge (MOW) osteotomy (Figure 2), in which bone chips from the resected lateral wedge are immediately grafted into the medial opening gap [1]. The common indications for the iVHTO procedure included (1) a knee with persistent pain due to medial osteoarthritis (OA) that did not improve despite 3 months of non‐operative therapy; (2) patients who want to return to active daily activities, sports, or labour; (3) a medial OA knee that needed a valgus correction angle >10°; (4) a medial OA knee with mild patellofemoral OA; (5) patients younger than 70 years. Contraindications involved (1) a loss of knee extension <10°, (2) a loss of knee flexion <120°, (3) a knee with a lateral meniscus injury requiring surgical treatment, (4) a knee with severe patellofemoral OA, (5) a knee with cruciate and collateral ligament insufficiency, (6) a history of infection in the knee or the tibia and (7) a history of severe trauma of the leg.

Among the 240 knees from 227 patients, surgeries were performed as follows: 113 knees (107 patients) by Surgeon A at Hospital I; 32 knees (31 patients) by Surgeon B at Hospital II; 64 knees (60 patients) by Surgeon C at Hospital III; and 31 knees (29 patients) by Surgeon D at Hospital I. Surgeon A had 45 years of overall clinical experience and 8 years of experience with this procedure; Surgeon B had 30 and 6 years, respectively; Surgeon C, 15 and 3 years; and Surgeon D, 10 and 1 year. All surgeries were performed in accordance with previously published technical reports [8, 26], and a standardised post‐operative rehabilitation protocol [8] was applied to all patients.

Follow‐up examinations were performed at the outpatient clinic at each hospital. For each patient, pre‐operative demographic data and intraoperative findings were obtained from medical records. Antero‐posterior radiographs of the knee and whole lower limb were taken to evaluate the HTO surgery preoperatively, 1 year postoperatively, and at the final follow‐up period. To evaluate the fibular osteotomy, the following variables were examined. (1) Post‐operative complications were detected from the medical record. (2) The period needed for complete union at the fibular osteotomy site was determined by evaluating the radiographs taken at 2, 3, 6, 9, 12 and 24 months and final follow‐up period after surgery using the previously reported radiographic scoring method [21]. (3) Clinical symptoms at the osteotomy site were examined in the outpatient clinic at the final follow‐up period. (4) The overall clinical outcomes of HTO surgery combined with fibular osteotomy were evaluated using the Japanese Orthopaedic Association (JOA) Score, which was the standard knee function scale in Japan [1, 21], Lysholm score [12], and Knee Injury and Osteoarthritis Outcome Score (KOOS) [16] at both the pre‐operative and final follow‐up periods.

### Fibular osteotomy procedures

The techniques of the AOOL procedure were previously reported in detail [26]. Briefly, a 4‐cm longitudinal incision was made on the skin at the lateral aspect of the leg, which was at the central portion between the fibular head and the lateral malleolus. A longitudinal incision was made on the central portion of the crural fascia. The central portion of the fibula was circumferentially isolated from the surrounding tissues using two curved retractors. An acute oblique osteotomy was performed on the fibular shaft in a quasi‐frontal plane inclined at 25°–30° to the long axis of the fibula, using a thin oscillating saw. Immediately before the osteotomy was completed, two thin holes were created with a 2‐mm thick Kirschner wire on the lateral surface of the fibula. Each of the two holes was located either proximally or distally, at a point 3 mm from the fibular osteotomy line (Figure 1a). Then a No. 2 polyester thread (Ethibond Excel Suture; Johnson & Johnson Medical N.V.) was passed through the two holes (Figure 1b). HTO was then performed. When the HTO was completed, the ends of the osteotomized fibula were displaced (Figure 1c). Then, the dislocated fibular ends were reduced using a thin elevator to ensure as much contact as possible between the osteotomy surfaces (Figure 1d), and the polyester thread was securely tied (Figure 1e). This ligation technique maintained contact between the fibular ends after surgery, leaving some degree of displacement and angulation. The subcutaneous tissue and the skin were closed after irrigation. There was no need to leave a suction tube connected to a drainage bag at the osteotomy site because post‐operative bleeding from this site was minimal.

### iVHTO procedure

The details of the iVHTO procedure have been previously reported [8]. In pre‐operative planning, lateral wedge resection angle was determined for each knee so that the mechanical axis in the corrected limb passed through a point on the lateral tibial plateau, which was 65% lateral to the medial edge of the tibial joint surface. Under C‐arm fluoroscopic guidance, a K‐wire was inserted at the apex of the V‐shaped osteotomy, perpendicular to the anterior surface of the proximal tibia. A Protractor Installed Wire Insertion Guide (Olympus Terumo Biomaterials Corp), in which the angle of the two sleeves was matched to the planned osteotomy angle, was attached to the apex K‐wire. Then, two guide K‐wires were inserted from the lateral side of the tibia, each through its respective sleeve, directed toward the apex wire. Bi‐plane tibial osteotomies were performed. First, a coronal ascending osteotomy was made using a thin oscillating saw, parallel to the anterior surface of the tibial tubercle. Next, a lateral closing hemi‐wedge osteotomy was performed along the two guide K‐wires, using a thin oscillating saw and a thin chisel. Finally, a single‐plane osteotomy for medial hemi‐wedge opening was made using a thin chisel, following the previously created parallel holes. These parallel holes were created in the tibia by inserting a 2‐mm diameter K‐wire through parallel sleeves of a Parallel Drill Guide (Olympus Terumo Biomaterials), which was mounted on the apex K‐wire. Then, the valgus tibial correction was made by making an incomplete fracture at the apex portion of the V‐shaped osteotomy, and temporary fixation was performed using two other K‐wires. Tibial fixation was performed using a locking compression plate (Olympus Terumo Biomaterials) installed at the lateral side, using the quantitative technique [6]. Finally, morselized bone chips prepared from the wedged bone resected from the lateral tibia were implanted into the medial opening space created by the lateral hemi‐wedge closing. After irrigation, the subcutaneous tissue and the skin were closed.

### Rehabilitation protocol

A previously reported rehabilitation protocol, which was created for the iVHTO [8], was used postoperatively. No specific rehabilitation for the fibular osteotomy was performed. Quadriceps exercise with quadriceps‐setting and straight leg raising was allowed 1 day after surgery. Passive knee motion from 0 to 90 was allowed during the first 2 weeks, and active knee motion from 0 to 120 was encouraged thereafter. Partial weightbearing with the aid of crutches was permitted at 3 weeks, and full weightbearing was allowed at 5 weeks.

### Radiological evaluations

To clarify the degree of alignment correction performed by iVHTO surgeries, intra‐operative correction angle as well as pre‐ and post‐operative lateral femorotibial angle (FTA), hip‐knee‐ankle angle (HKA), ratio of the point at which the mechanical axis passed across the joint line to the width of the tibial plateau (%MA), and medial‐proximal tibial angle (MPTA) values were measured on anteroposterior (AP) radiographs.

**Figure 3:**
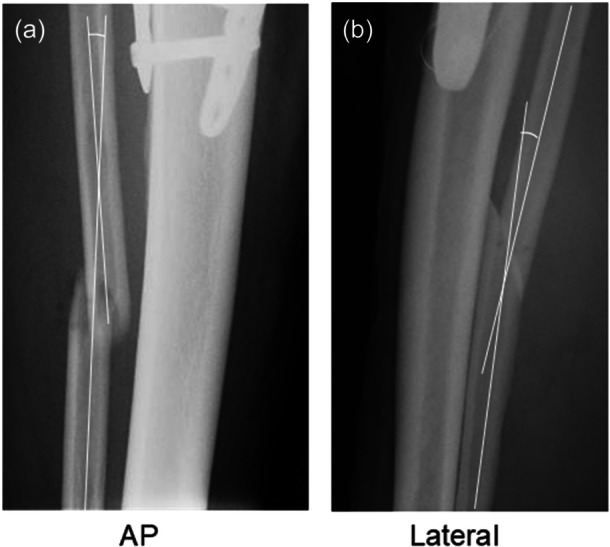
Anteroposterior (AP) and lateral radiographs of the fibula taken immediately after surgery. (a) Frontal angulation (valgus alignment). (b) Sagittal angulation (flexion alignment).

The radiographic union score for fibular osteotomy (RUSF) [21] was used to quantitatively evaluate bone union at the fibular osteotomy site on AP and lateral radiographs. On each set of AP and lateral radiographs, the shortest distance between the separated bone ends at the osteotomy site was defined as the 'initial osteotomy gap' [21]. The angles formed between the proximal and distal fibular shafts on AP and lateral radiographs were defined as the frontal and sagittal angulations of the fibular shaft, respectively (Figure 3). Positive values of frontal and sagittal angulations indicated valgus and flexion alignments, respectively.

**Figure 4:**
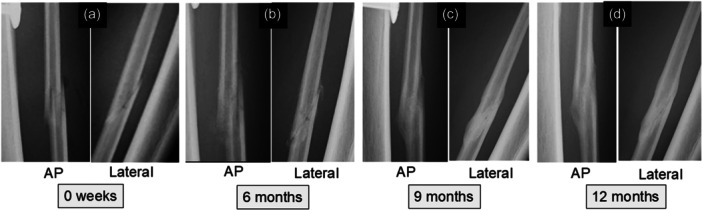
A representative patient (age, 52 years) who underwent the acute oblique osteotomy and ligation procedure. Anteroposterior (AP) and lateral radiographs of the fibular shaft were taken immediately after surgery (a), at 6 months (b), 9 months (c) and 12 months (d) postoperatively. As the radiographic union score for fibular osteotomy (RUSF) reached 12 points at 9 months postoperatively, the bone union time was determined to be 9 months.

The RUSF was created by modifying the radiographic union score for tibial fractures (RUST), which was recognised as the most reliable and repeatable outcome measure to evaluate radiographic union of tibial fractures [10, 24]. The intra‐ and interrater reliability (the weighted kappa) of the RUSF system was reported by the authors to be 0.977 and from 0.781 to 0.862, respectively, which were sufficient to evaluate the healing process at the fibular osteotomy site [21]. In the RUSF, healing of the fibular osteotomy site was assessed at each of the medial, lateral, anterior, and posterior cortices visible on AP or lateral radiographs. Each cortex was given a score of 1 point if an osteotomy line was clearly visible with no callus, 2 points if there was callus formation but an osteotomy line was still visible, and 3 points if the cortices were completely bridged by callus formation but an osteotomy line was still visible or if there was a bridging callus with no evidence of an osteotomy line. The individual scores for the four cortices were summed to give a total score at each period. In the present study, a maximum score of 12 points on the RUSF was considered to indicate complete union, and the time when a total score reached 12 points was referred to as union time at the fibular osteotomy site for each patient (Figure 4). When the osteotomy line was still clear at the osteotomy site 12 months after surgery, this was considered to indicate non‐union at the osteotomy site [19]. Two experienced orthopaedic surgeons (D.S. and J.O.), who were blinded to all clinical information, served as observers and independently scored each set of radiographs. The mean score of the two observers was used as a measured value.

### Clinical evaluations

Post‐operative complications were detected from the electronic medical record system. To assess residual clinical symptoms at the fibular osteotomy site, each patient was asked at the final follow‐up examination about lateral leg pain during walking and tenderness at the osteotomy site. The overall clinical evaluations of symptoms and knee functions were evaluated in each patient using JOA Score [1, 21], Lysholm score [12] and KOOS [16].

### Statistical analysis

In this study, surgical treatments were performed by four surgeons. Therefore, before pooling all cases into a single cohort, it was verified that patient groups across the four surgeons showed no significant differences with respect to patient characteristics, procedural variations, and clinical outcomes. This assessment was not intended to evaluate equivalence or non‐inferiority among the surgeons. Comparisons of continuous data among the four surgeons were performed using one‐way analysis of variance (ANOVA), followed by Fisher's protected least significant difference test for post hoc multiple comparisons. The pre‐ and post‐operative data were compared using the paired t‐test. Normality was assessed using the Shapiro–Wilk test to confirm the appropriateness of parametric testing. Comparisons of categorical variables among the four surgeons were performed using Pearson's chi‐square test or Fisher's exact test, as appropriate. To present the complication and non‐union rates, two‐sided exact 95% confidence intervals (CIs) for the proportions were calculated using the Clopper–Pearson method.

For the logistic regression analyses, the time to bone union at the fibular osteotomy site was dichotomised at the median value of 6 months. Union times of 6 months or less were classified as Category E (early healing), and those exceeding 6 months as Category L (late healing). First, univariate logistic regression analyses were performed to evaluate the association between the fibular union time and potential explanatory variables, including gender, age, body mass index (BMI), bone mineral density (BMD), Kellgren–Lawrence (KL) grade, the correction angle of the tibia during HTO, and the initial gap and angulation at the fibular osteotomy site. Secondly, a multivariate logistic regression analysis was performed using the fibular union time (Categories E and L) as the dependent variable and the significant factors identified in the univariate logistic regression analyses as potential explanatory variables. Results are presented as regression coefficients (*β*) with standard errors (SE), odds ratios (ORs) with 95% CIs, and *p*‐values.

Statistical analyses were performed using IBM SPSS Statistics (version 26; IBM, Armonk, NY, USA). The significance level was set at *p* = 0.05.

## RESULTS

### Demographic data

**Figure 5:**
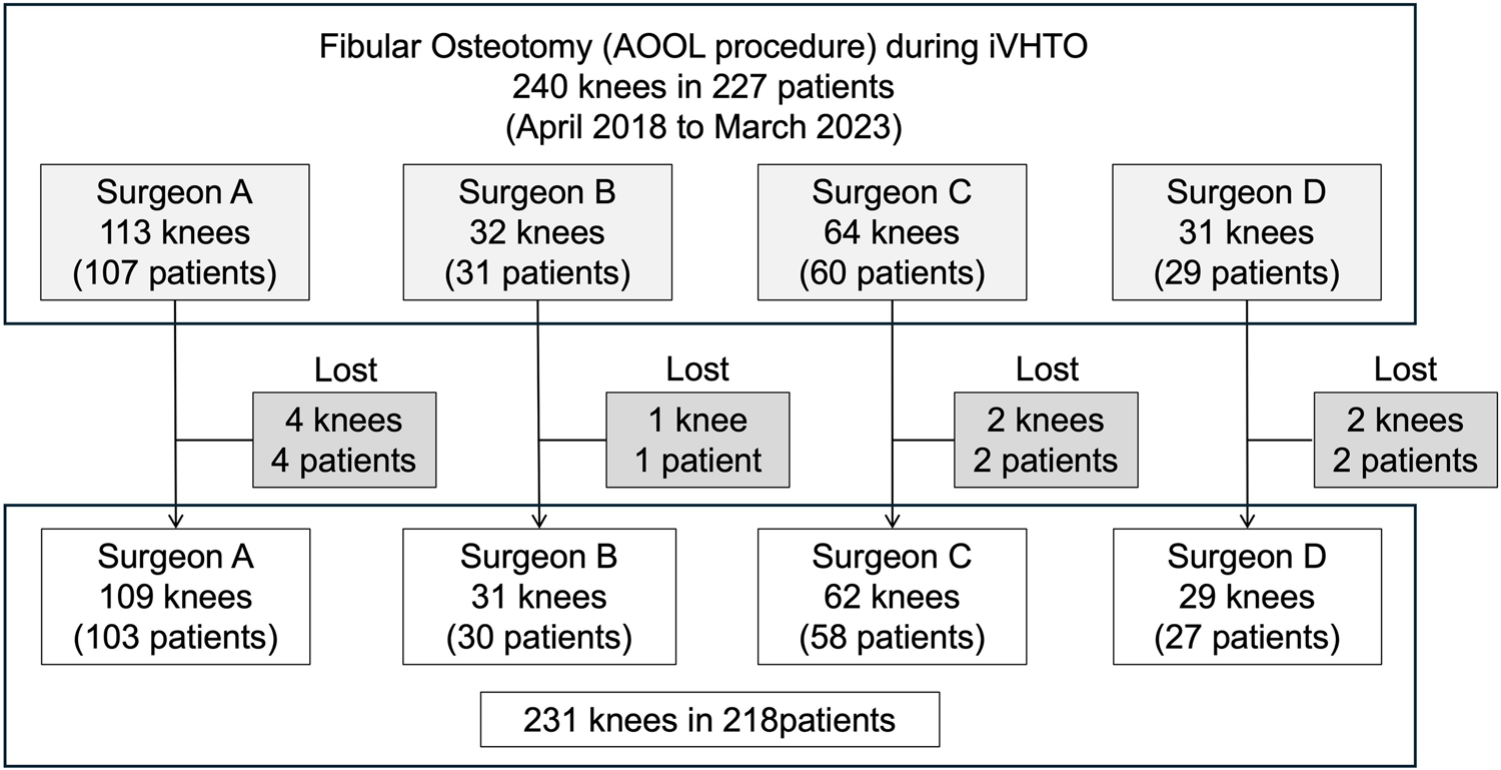
Flowchart of the patients in the study groups. AOOL, acute oblique osteotomy and suture ligation procedure; iVHTO, inverted V‐shaped high tibial osteotomy.

Among the 227 patients (240 knees) who underwent fibular osteotomy with AOOL procedure, nine patients (9 knees) were lost to follow‐up. The follow‐up rate was 96.3%. Subsequently, a total of 231 knees of 218 patients (115 men and 103 women) were enroled in the present study. Of the 231 knees, 109 knees (105 patients), 62 knees (55 patients), 31 knees (30 patients) and 29 knees (28 patients) were operated by Surgeons A, B, C and D, respectively (Figure 5).

The follow‐up period ranged from 2 to 4 years. The mean age was 62.1 years (range, 35–75 years). The mean BMI was 25.6 kg/m^2^ (range 18.6–43.3). Regarding the Kellgren–Lawrence (K‐L) OA grade, 9, 45, 82 and 83 knees indicated Grade 1, 2, 3 and 4, respectively (Table 1). Among the four surgeons, no significant differences were observed in terms of age, sex, height, body weight, BMI, bone mineral density and K‐L grade (Table 1).

**Table 1:** Demographic data of the patients, compared among Surgeons A, B, C and D.

	Total	A	B	C	D	*p*‐value
Number of knees (patients)	231 (218)	109 (105)	31 (30)	62 (55)	29 (28)	–
Age (years)	62.1 (8.2)	60.9 (8.3)	60.2 (6.3)	64.0 (8.4)	64.3 (8.1)	0.35
Male/female (patients)	113/105	58/47	15/15	25/30	14/14	0.55
Right/left (knees)	111/120	50/59	11/20	37/25	13/16	0.13
Height (cm)	161.2 (9.4)	162.1 (8.7)	164.0 (11.9)	158.2 (8.9)	161.4 (9.0)	0.55
Body weight (kg)	66.8 (12.6)	66.9 (12.5)	67.6 (13.0)	65.1 (11.5)	69.5 (15.0)	0.74
Body mass index (kg/m²)	25.6 (3.7)	25.3 (3.9)	25.0 (3.2)	25.8 (3.4)	26.5 (4.3)	0.37
Bone mineral density (%)	93.4 (15.1)	93.5 (14.9)	94.3 (14.6)	93.6 (16.8)	92.0 (13.9)	0.91
OA grade Grade 1 (KL classification)	9 (3.8%)	7 (6.4%)	2 (6.5%)	0 (0%)	0 (0%)	0.14
Grade 2	45 (19.4%)	25 (22.9%)	7 (22.5%)	7 (11.3%)	6 (20.6%)	
Grade 3	82 (35.4%)	36 (33.0%)	10 (32.2%)	21 (33.8%)	15 (51.7%)	
Grade 4	83 (35.9%)	38 (34.8%)	11 (35.4%)	27 (43.5%)	7 (24.1%)	

*Note*: Bone mineral density was shown as the rate (%) to the young adult mean. The continuous data are reported as ‘the mean (the standard deviation)’.

Abbreviations: KL, Kellgren–Lawrence classification; MM, medial meniscus; OA, osteoarthritis; OAT, osteochondral autograft transfer.

### Operative details of HTO

No significant differences were observed in each alignment parameter among the four surgeons (Table 2). In the 231 knees analysed, the mean correction angle was 11.7°. The mean MPTA increased significantly from 83.7° preoperatively to 92.9° postoperatively (*p* < 0.0001). Consequently, all post‐operative alignment parameters showed significant changes compared to the pre‐operative values. The post‐operative %MA was 64.1%, closely approximating the target value.

**Table 2:** Details of the HTO surgeries performed by Surgeons A, B, C, D.

	Total	A	B	C	D	*p*‐value
Number of knees	231	109	31	62	29	–
Pre‐operative						
FTA (degrees)	180.8 (3.1)	180.8 (3.0)	181.8 (2.2)	180.5 (3.3)	181.4 (3.7)	0.29
HKA (degrees)	−7.0 (2.8)	−7.1 (2.6)	−6.4 (2.5)	−6.7 (2.8)	−7.7 (3.5)	0.46
%MA (%)	18.1 (12.5)	17.8 (11.7)	18.9 (11.5)	19.8 (11.6)	14.4 (17.2)	0.24
MPTA (degrees)	83.7 (2.1)	83.8 (2.0)	83.1 (1.9)	84.0 (2.3)	83.5 (2.2)	0.80
Intraoperative						
Correction angle (degrees)	11.7 (2.4)	11.8 (2.3)	11.0 (2.2)	12.2 (2.3)	11.0 (2.8)	0.47
Post‐operative						
FTA (degrees)	170.6* (2.7)	170.4* (2.7)	170.5* (2.2)	170.8* (2.6)	171.4* (3.2)	0.10
HKA (degrees)	3.4* (2.8)	3.5* (2.9)	3.7* (1.6)	3.3* (3.0)	2.9* (2.9)	0.071
%MA (%)	64.1* (11.0)	64.9* (11.3)	63.6* (7.6)	64.7* (11.6)	60.1* (12.3)	0.14
MPTA (degrees)	92.9* (2.3)	93.0* (2.2)	92.6* (2.4)	92.9* (2.6)	93.1* (2.9)	0.25

*Note*: The data are shown as 'the mean (the standard deviation)'. Additionally, the effect of iVHTO on each item was evaluated by comparing the pre‐ and post‐operative data (an asterisk indicates *p* < 0.001*).

Abbreviations: FTA, lateral femorotibial angle; HKA, hip‐knee‐ankle angle; MPTA, medial‐proximal tibial angle; %MA, ratio of the point at which the mechanical axis passed across the joint line to the width of the tibial plateau.

### Operative details of fibular osteotomy

No significant differences were observed in the initial osteotomy gap or the initial fibular angulation among the four surgeons (Table 3). The initial osteotomy gap averaged 0.6 mm, with the standard deviation (SD) of 1.1. The initial fibular angulation averaged 4.0°, with the SD of 3.5 in the anteroposterior view (Table 3).

**Table 3:** Details of the fibular osteotomies performed by Surgeons A, B, C, D.

	Total	A	B	C	D	*p*‐value
Initial gap (mm)	0.6 (1.1)	0.6 (1.0)	0.4 (1.0)	0.5 (1.0)	1.1 (1.3)	0.053
Initial angulation (degrees)						
Frontal (AP view)	4.1 (3.5)	3.9 (3.2)	4.0 (3.5)	3.7 (3.6)	5.5 (4.0)	0.121
Sagittal (lateral view)	0.6 (3.0)	0.8 (2.8)	0.2 (2.4)	0.4 (3.5)	0.8 (2.9)	0.621
Angulation at the time of bone union (degrees)						
Frontal (AP view)	2.7 (2.6)	2.7 (2.2)	2.9 (3.0)	2.1 (3.0)	3.6 (2.8)	0.092
Sagittal (lateral view)	0.6 (2.1)	0.6 (2.1)	0.3 (2.1)	0.5 (2.4)	0.8 (1.9)	0.300

*Note*: The data are shown as 'the mean (the standard deviation)'.

Abbreviations: AP, anteroposterior; Initial gap, initial osteotomy gap.

### Complications

**Figure 6:**
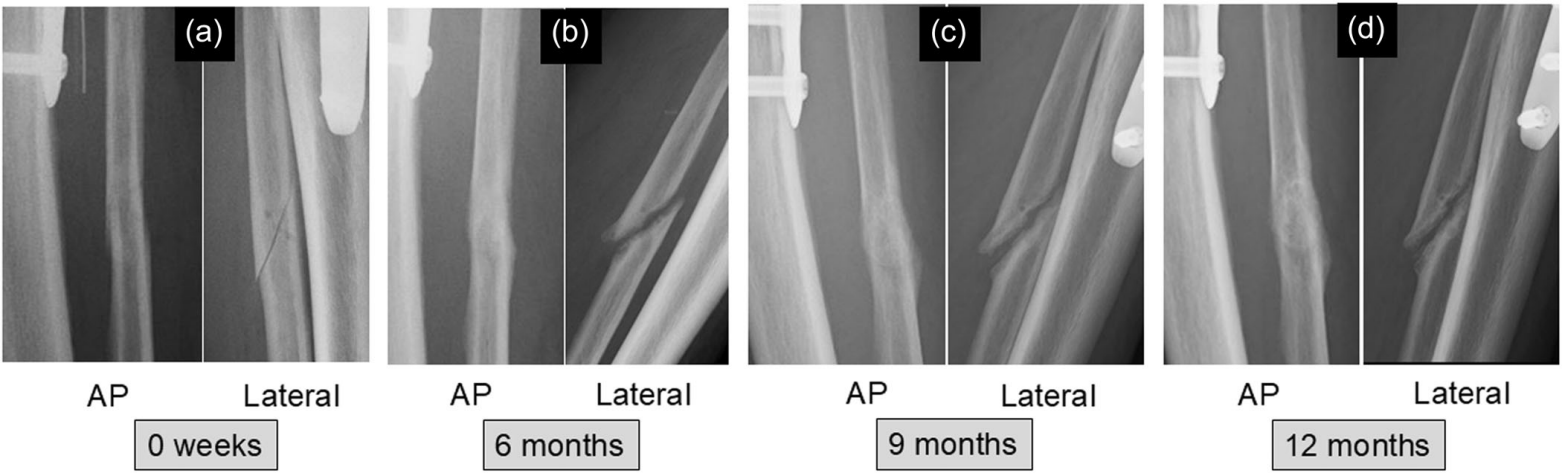
A 58‐year‐old woman with asymptomatic non‐union. The anteroposterior (AP) and lateral radiographs taken immediately after surgery show that adequate contact between the osteotomy ends was achieved during surgery. However, callus formation remained insufficient throughout the post‐operative period.

Of all 231 fibulas, there were four cases with post‐operative complications around the fibular osteotomy site, including two cases of asymptomatic non‐union (Figure 6), one case of symptomatic delayed union, and one case of asymptomatic peroneal muscle hernia. The complication rate was 1.7%, with an exact 95% CI of 0.5%–4.4% (Table 4). No significant differences were observed among the four surgeons (Table 4).

**Table 4:** Post‐operative bone union and complications.

	Total	A	B	C	D
Number of knees	231	109	31	62	29
Cases of complication	4	3	0	1	0
Complication rate (exact 95% CI)	1.7% (0.5–4.4)	2.8% (0.6–7.8)	0.0% (0.0–11.2)	1.6% (0.04–8.6)	0.0% (0.0–11.9)
Cases of non‐union	3	2	0	1	0
non‐union rate (exact 95% CI)	1.3% (0.3–3.7)	1.8% (0.2–6.4)	0.0% (0–11.2)	1.6% (0.04–8.6)	0.0% (0–11.9)
Time to bone union; months (SD)	7.0 (2.4)	6.8 (2.3)	7.3 (2.6)	6.8 (3.1)	7.2 (2.2)

*Note*: A, B, C, and D show the four surgeons. Continuous data are shown as ‘the mean (the standard deviation [SD])’. Exact (Clopper–Pearson) 95% confidence intervals (CI) are shown for proportions.

### Bone healing at the fibular osteotomy site

No significant differences were found in the individual non‐union rate or the time to complete bone union at the fibular osteotomy site among the four surgeons (Table 4). Of all 231 fibulas, there were three non‐union fibulas of three patients. The non‐union rate was 1.3%, with an exact 95% CI of 0.3%–3.7% (Table 4). Accordingly, the bone union rate was 98.7%, with an exact 95% CI of 96.3%–99.7%. Among the 228 fibulas that achieved complete union at the final follow‐up, the average time to union was 7.0 months, with SD of 2.4 (Table 4).

### Clinical outcomes

No significant differences were observed in pre‐ and post‐operative JOA, Lysholm or KOOS scores among the four surgeons (Table 5). The overall post‐operative JOA and Lysholm scores were significantly improved compared to the pre‐operative scores (*p* < 0001). Each post‐operative KOOS subscale score also showed a significant improvement compared to its respective pre‐operative value (*p* < 0001) (Table 5).

**Table 5:** Functional and patient‐reported outcomes.

	Total (231)	A (109)	B (31)	C (62)	D (29)	*p*‐value
Pre‐operative						
JOA score (points)	66.9 (10.6)	67.6 (10.5)	66.2 (13.4)	67.7 (9.9)	63.2 (8.5)	0.23
Lysholm score (points)	59.8 (15.5)	59.4 (15.0)	60.8 (19.1)	61.2 (14.6)	57.6 (15.6)	0.43
KOOS pain (points)	55.3 (18.9)	54.7 (19.2)	57.7 (17.4)	56.4 (17.9)	53.1 (18.0)	0.64
Symptom	60.6 (19.9)	59.8 (21.2)	59.8 (14.1)	62.5 (21.8)	60.7 (19.3)	0.97
Activity of daily living	71.4 (14.4)	71.1 (15.6)	72.1 (13.7)	72.4 (14.5)	69.9 (14.9)	0.48
Sports/recreation	32.4 (21.2)	32.9 (21.8)	32.2 (25.4)	33.3 (18.8)	30.0 (19.4)	0.15
Quality of life	32.6 (19.0)	33.1 (19.6)	31.4 (17.8)	32.1 (18.9)	33.1 (19.3)	0.99
Post‐operative						
JOA score (points)	91.6* (5.3)	91.1* (5.7)	93.5* (5.4)	91.3* (4.6)	91.2* (5.2)	0.88
Lysholm score (points)	93.0* (6.0)	93.5* (6.1)	93.8* (6.9)	93.0* (5.0)	91.4* (6.4)	0.35
KOOS pain (points)	85.5* (12.6)	86.1* (12.0)	88.4* (10.7)	83.6* (12.1)	84.9* (15.0)	0.64
Symptom	83.6* (12.3)	83.7* (12.6)	85.4* (9.5)	81.5* (13.7)	85.5* (13.3)	0.77
Activity of daily living	89.0* (9.4)	89.3* (9.2)	90.1* (9.0)	87.2* (9.9)	90.0* (9.8)	0.92
Sports/recreation	63.0* (23.4)	62.9* (23.8)	63.6* (23.0)	61.2* (23.3)	65.9* (23.8)	0.88
Quality of life	67.6* (19.7)	67.4* (19.7)	66.7* (19.8)	64.5* (20.0)	74.7* (18.3)	0.28

*Note*: The data are shown as 'the mean (the standard deviation)'. Additionally, the effect of the surgery on each item was evaluated by comparing the pre‐ and post‐operative data (an asterisk indicates *p* < 0.001*).

Abbreviations: JOA, Japanese Orthopaedic Association; KOOS, Knee Injury and Osteoarthritis Outcome Score.

### Factors associated with a prolonged time to fibular union

Univariate logistic regression analyses showed that time to bone union at the fibular osteotomy site was significantly associated with gender (OR 0.504; *p* = 0.034), BMD (OR 0.974; *p* = 0.045), OA grade (OR 1.921; *p* = 0.002) and the initial osteotomy gap (OR 1.363; *p* = 0.017) (Table 6). In contrast, age, BMI, the correction angle during HTO and the initial fibular angulation were not significantly associated with bone union time.

**Table 6:** Univariate logistic regression analyses were performed to evaluate the association between the time to complete bone union at the fibular osteotomy site (categories E and L) and each of the potential explanatory variables.

Potential explanatory variables	*β* (SE)	OR (95% CI)	*p*‐value
Gender (female vs. male)	−0.684 (0.324)	0.504 (0.263–0.942)	0.034*
Age (per 1 year)	0.022 (0.019)	1.022 (0.983–1.064)	0.266
BMI (per 1 kg/m²)	0.070 (0.039)	1.073 (0.992–1.162)	0.075
BMD (per 1 point)	−0.025 (0.012)	0.974 (0.949–0.998)	0.045*
KL grade (per +1 grade)	0.652 (0.216)	1.921 (1.278–2.991)	0.002*
Correction angle (per 1°)	0.120 (0.065)	1.128 (0.993–1.287)	0.066
Initial gap (per 1 mm)	0.309 (0.130)	1.363 (1.055–1.769)	0.017*
Fibula angulation A‐P (per 1°)	−0.008 (0.044)	0.991 (0.906–1.081)	0.848
Fibula angulation lateral (per 1°)	0.030 (0.052)	1.031(0.930–1.145)	0.562

*Note*: Each row shows the regression coefficient (*β*) with standard error (SE), odds ratio (OR) with 95% confidence interval (CI) and *p*‐value for each potential explanatory variable. *Statistically significant.

Abbreviations: BMD, bone mineral density; BMI, body mass index; Initial gap, initial osteotomy gap; KL grade, Kellgren–Lawrence osteoarthritis grade; YAM, Young Adult Mean.

Then, multivariable logistic regression analysis demonstrated that gender (OR 0.388; *p* = 0.0109), BMD (OR 0.950; *p* = 0.0010), OA grade (OR 2.145; *p* = 0.0013), and the initial osteotomy gap (OR 1.487; *p* = 0.0086) were independent variables significantly associated with the bone union time (Table 7). Namely, gender (male), lower BMD, higher OA grade and greater initial osteotomy gap were significant factors delaying bone union at the fibular osteotomy site.

**Table 7:** A multivariate logistic regression analysis was performed using the fibular union time (categories E and L) as the dependent variable and the significant factors identified in the univariate logistic regression analyses as potential explanatory variables.

Explanatory variables	*β* (SE)	OR (95% CI)	*p*‐value
Sex (female vs. male)	−0.946 (0.372)	0.388 (0.183–0.792)	0.0109*
YAM (per 1 point)	−0.051 (0.016)	0.950 (0.920–0.978)	0.0010*
KL grade (per +1 grade)	0.763 (0.237)	2.145 (1.376–3.497)	0.0013*
Initial gap (per 1 mm)	0.397 (0.151)	1.487 (1.105–2.006)	0.0086*

*Note*: Each row shows the regression coefficient (*β*) with standard error (SE), odds ratio (OR) with 95% confidence interval (CI) and *p*‐value for each potential explanatory variable. *Statistically significant.

Abbreviations: BMD, bone mineral density; BMI, body mass index; Initial gap, initial osteotomy gap; KL grade, Kellgren–Lawrence osteoarthritis grade; YAM, Young Adult Mean.

## DISCUSSION

The important findings in this study with the 231 fibular osteotomies were as follows. First, the complication rate was 1.7%, with an exact 95% CI of 0.5%–4.4%. There were no serious complications such as peroneal nerve palsy, infection, or blood vessel injuries. Second, the non‐union rate was 1.3%, with an exact 95% CI of 0.3%–3.7%. Accordingly, the bone union rate was 98.7%, with an exact 95% CI of 96.3%–99.7%. Thirdly, among the 228 fibulas that achieved union, the time to bone union averaged 7.0 months, with the standard deviation of 2.4. Fourth, logistic regression analyses indicated that gender (male), lower BMD, higher OA grade and greater initial osteotomy gap were significant factors delaying bone union at the fibular osteotomy site.

One limitation of this study is the absence of a control group treated with techniques other than the AOOL procedure. Therefore, direct comparisons of complication and bone union rates between the AOOL procedure and other fibular shortening techniques could not be performed in this study. In previous studies on fibular mid‐shaft resection procedures, the precise complication rate has not been reported, but peroneal nerve palsy and non‐union have been reported in 2.8%–13.7% [9, 15] and 14%–65% [3, 9, 15] of cases, respectively. In contrast, the present study demonstrated that the exact 95% CI was 0.5%–4.4% for the complication rate and 0.3%–3.7% for the non‐union rate. There were no cases of peroneal nerve palsy. These findings suggest that the AOOL procedure can achieve lower complication and higher bone union rates compared with the conventional procedures. However, direct comparative studies should be conducted to confirm this suggestion in the future.

On the other hand, it is difficult to compare the complication rate of the AOOL procedure with that of fibular head manipulation procedures, as the types of complications differ between them. For example, progression of PTFJ arthritis and increased lateral instability of the knee have been reported as new complications following PTFJ release [5, 14, 18, 20]. More importantly, Sanchez‐Soler et al. [18] reported that valgus correction of the HKA was often insufficient in LCWHTO combined with fibular head manipulation procedures, due to the limited fibular shortening effect of these procedures. This should be recognised as a serious concern regarding fibular head manipulation procedures, as achieving the planned correction of lower limb alignment is essential in HTO. In contrast, the present study using the AOOL procedure demonstrated a post‐operative %MA of 64.1%, which closely approximated the target value. This result indicates that cases of insufficient correction were rare. This finding suggests that the AOOL procedure allows sufficient fibular shortening during LCWHTO, which represents an advantage over fibular head manipulation procedures.

It is well known that fibular shaft fractures have a high healing capacity because this region is surrounded by muscle tissues and receives abundant blood supply from the nutrient arteries [23]. According to Bhadra et al. [2], fibular mid‐shaft fractures show a spontaneous union rate of about 90%, although the time to complete union varies widely. In the present study, the exact 95% CI for the bone union rate was 96.3%–99.7%. This finding suggests that the AOOL procedure does not impair the fibula's inherent healing capacity. However, the mean time to bone union following the AOOL procedure was relatively prolonged, with an average of 7.0 months (SD, 2.4). The mean time to bone union after iVHTO in the same cohort was reported to be 12.4 months (SD, 4.4) [25]. Therefore, fibular union after the AOOL procedure occurred considerably later than tibial union after iVHTO. Ideally, it may be preferable for patients if fibular healing takes place at a timing similar to that of tibial healing at the osteotomy site in HTO. Therefore, future studies are required to enhance healing at the fibular osteotomy site. In this study, as an initial step toward that goal, factors affecting healing at the fibular osteotomy site were elucidated. Namely, gender (male), lower BMD, higher OA grade and greater initial osteotomy gap were significant factors delaying bone union at the fibular osteotomy site. Among these factors, only the initial osteotomy gap is a technical factor. Bone healing at the fibular osteotomy site may be accelerated if the initial osteotomy gap is reduced by improving the suture ligation technique in the AOOL procedure. Further research is warranted to develop a simple, safe, and cost‐effective method for minimising the initial osteotomy gap in the near future.

The rarity of post‐operative complications with the AOOL procedure may be attributed to three technical features of the technique. First, the fibular osteotomy was carried out at the central portion of the fibular shaft, which had been reported to be the safest site for avoiding peroneal nerve palsy [4, 7, 17]. Second, the surgical technique was simple, contributing to a shortened operation time, which has been reported to be approximately 8 min [21]. Third, the two curved retractors can isolate the anterior, posterior, and medial surfaces of the fibula from the surrounding neurovascular tissues when the fibular osteotomy was performed.

In Table 3, the *p*‐value for the initial gap was 0.053, approaching statistical significance. This may be attributed to the fact that the values for Surgeon D differed relatively from those of the other three surgeons. This surgeon had the least surgical experience, which may have contributed to the tendency for their results to differ from those of the other surgeons. In the present study, however, no remarkable increase in complications or non‐union was observed even for that surgeon.

In the present study, clinical outcomes were assessed using the JOA score, Lysholm score, and KOOS subscale scores, because no established scoring system exists for evaluating fibular osteotomy associated with HTO. The scoring systems used in this study are not designed to directly evaluate the isolated clinical outcomes of fibular osteotomy. However, if fibular osteotomy results in complications or painful symptoms, it may impair walking and activity, thereby lowering these scores. Therefore, the scoring systems may be able to reflect clinical outcomes following fibular osteotomy.

The present study has several strengths. First, this study enroled 231 knees, which is a sufficiently large sample size to evaluate a surgical procedure. Secondly, all patients underwent identical surgery and post‐operative management according to the published protocol [8]. Thirdly, bone union at the osteotomy site was evaluated using a quantitative scoring system with high intra‐ and inter‐rater reliability [21].

In the present study, however, several limitations should be noted. First, as noted in the preceding paragraph, the study lacked a control group treated with techniques other than the AOOL procedure. Second, the observation period in this study ranged from 2 to 4 years. Therefore, the long‐term follow‐up results at the fibular osteotomy site remain unclear. However, previous reports indicate that nearly all complications following fibular osteotomy occur within 2 years after surgery [3, 9, 14, 15, 18, 20]. Therefore, this study is considered to have captured all complications arising during the acute and subacute post‐operative phases associated with the AOOL procedure. Thirdly, surgeon experience is heterogeneous not only in years but also in case volume. Fourthly, the results of the AOOL procedure have not been evaluated in various LCWHTO techniques other than the iVHTO procedure. These limitations may influence the conclusions. However, the present study may provide valuable new information into the field of fibular osteotomy for LCWHTO.

In conclusion, among the 231 fibular osteotomies performed using the AOOL procedure, complications were rare, and the bone union rate was remarkably high. These results suggest that the AOOL procedure may be a useful fibular shortening technique prior to LCWHTO. However, the average time to union was 7 months, which is considered relatively prolonged. Gender (male), lower BMD, higher OA grade and greater initial osteotomy gap were significant factors delaying bone union at the fibular osteotomy site.

## AUTHOR CONTRIBUTIONS

Dai Sato collected the data, made the analysis, and drafted the work. Kazunori Yasuda as conducted this study and completed the draft. Eiji Kondo, Shuken Kai and Koji Yabuuchi performed the surgery and recruiting patients. Jun Onodera supported the statistical analysis. Norimasa Iwasaki and Tomonori Yagi advised on the interpretation of the data.

## CONFLICT OF INTEREST STATEMENT

The authors declare no conflicts of interest.

## ETHICS STATEMENT

Ethical approval for this study was obtained from the institutional review board of Yagi Orthopaedic Hospital (approval No. H28‐0001). The requirement for informed consent was waived by the institutional review board because this study used anonymized retrospective data.

## Supporting information

Supporting File

## Data Availability

The data sets that support the findings of this study are available on request from the corresponding author. The data are not publicly available due to patient privacy regulations and institutional ethical restrictions.
